# Optimal monitor positioning and camera rotation angle for mirror image: overcoming reverse alignment during laparoscopic colorectal surgery

**DOI:** 10.1038/s41598-019-44939-0

**Published:** 2019-06-10

**Authors:** Susumu Miura, Taro Oshikiri, Yukiko Miura, Gosuke Takiguchi, Nobuhisa Takase, Hiroshi Hasegawa, Masashi Yamamoto, Shingo Kanaji, Yoshiko Matsuda, Kimihiro Yamashita, Takeru Matsuda, Tetsu Nakamura, Satoshi Suzuki, Yoshihiro Kakeji

**Affiliations:** 1 Division of Gastrointestinal Surgery, Department of Surgery, Graduate School of Medicine, Kobe University, 7-5-2, Kusunoki-cho, Chuo-ku, Kobe, Hyogo 650-0017 Japan; 2 Division of Minimally Invasive Surgery, Department of Surgery, Graduate School of Medicine, Kobe University, 7-5-2, Kusunoki-cho, Chuo-ku, Kobe, Hyogo 650-0017 Japan; 3 Division of Community Medicine and Medical Network, Department of Social Community Medicine and Health Science, Graduate School of Medicine, Kobe University, 7-5-2, Kusunoki-cho, Chuo-ku, Kobe Hyogo, 650-0017 Japan

**Keywords:** Colon, Rectum

## Abstract

Mirror image is one of the most difficult situations that the assistant surgeon encounters in laparoscopic colorectal surgery. The aim of the present study was to investigate whether task performance with mirror images improves by changing the position of the monitor and the rotation angle of the camera. Twenty-four surgeons performed the task under different conditions: Coaxial image (C), Mirror image (M), Mirror image + Monitor on the left side of participants (M + Mon), Mirror image + Camera rotated 90 degrees to the right (M + Cam), and Mirror image + Monitor on the left side + Camera rotated to the right (M + Mon + Cam) in a training box. The outcome measure was the mean time for completing the task. The mean time for completing the task, in decreasing order, was M (111.4 ± 58.9 seconds) > M + Mon (70.5 ± 29.4 seconds) > M + Cam (47.1 ± 17.1 seconds) > M + Mon + Cam (33.4 ± 10.3 seconds) > C (20.5 ± 3.5 seconds). (multivariable analysis of variance (MANOVA), *p* = 7.9 × 10^−7^) Task performance with mirror images improved by changing the monitor positioning and camera rotation angle. This novel method is a simple way to overcome mirror image in laparoscopic colorectal surgery.

## Introduction

In laparoscopic surgery, the position of the surgeon, working ports, camera, and monitor influence task performance^1–4^. Ideally, the camera should face the target in the direction of the surgeon’s line of sight, and the monitor should be placed in front of the surgeon^1–4^. The best situation is called a coaxial image. However, it is extremely difficult for the surgeon to perform laparoscopic tasks if the camera is placed on the surgeon’s opposite side. The worst situation is called a mirror image (reverse alignment)^1,2^.

Mirror image is one of the most difficult situations encountered by the assistant surgeon in laparoscopic sigmoidectomy or anterior resection (left-sided colorectal surgery)^5^. The assistant usually encounters a mirror image during lymph node dissection around the inferior mesenteric artery (IMA) because the assistant stands on the opposite side of the camera.

Surgical skills when working with mirror images could improve with laparoscopic experience and training^5–8^. However, participation in at least 30–40 laparoscopic colorectal surgeries is needed for the assistant to gain surgical skills for working with a mirror image^5^. It is difficult for young surgeons to participate in a sufficient number of cases^6^. It might be traumatic and time-consuming for an inexperienced assistant to support an operative field.

Several studies have suggested that rotating the view on the monitor for the assistant by 180 degrees (left-right reversed and upside-down image) with the image converter improves task performance with mirror images^9–12^. However, most facilities do not have an image converter, which is an expensive piece of equipment. Simply turning the camera 180 degrees could rotate the view on the monitor by 180 degrees^9^. However, the surgeon might become confused, because the surgeon’s view on the monitor is also rotated by 180 degrees. The aim of the present study was to evaluate whether performance with mirror images improves with changes in monitor positioning and camera rotation angle.

## Results

**Figure 1:**
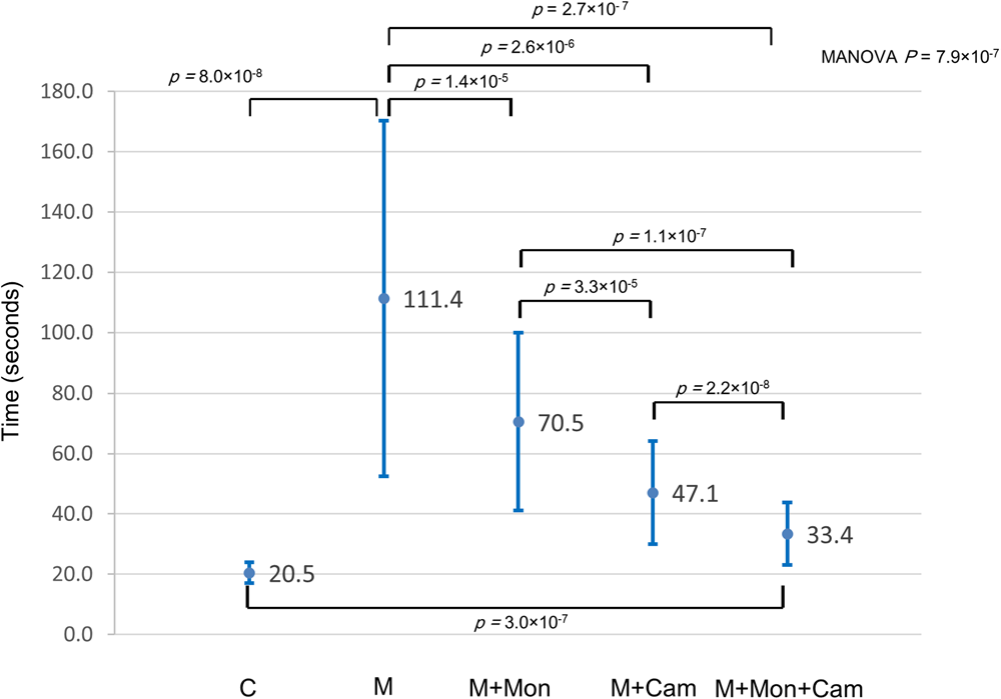
Duration of the modified peg transfer task. Plotting the time (mean ± standard deviation, in seconds) needed to complete the modified peg transfer task for five conditions.

Table 1 and Fig. 1 showed the time for completing the task under each of the five conditions. The Shapiro-Wilk test revealed that the time under each of the five conditions was approximately normally distribution. The mean time for completing the task with a mirror image (111.4 ± 58.9 seconds) was significantly longer than with a coaxial image (20.5 ± 3.5 seconds) (M vs. C, paired t test, *p* = 8.0 × 10^−8^). The mean time for completing the task when the monitor was placed on the left side of the participant with a mirror image (70.5 ± 29.4 seconds) was significantly shorter than with a mirror image alone (111.4 ± 58.9 seconds) (M + Mon vs. M, paired t test, *p* = 1.4 × 10^−5^). The mean time for completing the task when the camera was rotated 90 degrees to the right (47.1 ± 17.1 seconds) was significantly shorter than with a mirror image alone (111.4 ± 58.9 seconds) (M + Cam vs. M, paired t test, *p* = 2.*6* × *10*^−*6*^). The mean time for completing the task in condition M + Cam (47.1 ± 17.1 seconds) was significantly shorter than in condition M + Mon (70.5 ± 29.4 seconds) (M + Mon vs. M + Cam, paired t test, *p* = 3.3 × 10^−5^).

**Table 1:** The time for completing the task under each condition.

	C	M	M + Mon	M + Cam	M + Mon + Cam
Mean time (seconds)	20.5	111.4	70.5	47.1	33.4
Standard deviation	3.5	58.9	29.4	17.7	10.3
Max	14.0	19.8	20.8	16.7	15.8
Min	26.5	250.4	121.2	83.0	55.7
*P-*value (Shapiro-Wilk test)	0.39	0.51	0.54	0.77	0.57

The mean time for completing the task when the monitor was placed on the left side of the participant and the camera was rotated 90 degrees to the right with a mirror image (33.4 ± 10.3 seconds) was the shortest (M + Mon + Cam vs. M + Cam, paired t test, *p* = *2.2* × *10*^−*8*^; M + Mon + Cam vs. M + Mon, paired t test, *p* = *1.1* × *10*^−*7*^). The mean time for completing the task in condition M + Mon + Cam (33.4 ± 10.3 seconds) was still significantly longer than the mean time for completing the task with a coaxial image (20.5 ± 3.5 seconds) (M + Mon + Cam vs. C, paired t test, *p* = 3.0 × 10^−7^). In summary, the mean time for completing the task, in decreasing order, was M (111.4 ± 58.9 seconds) > M + Mon (70.5 ± 29.4 seconds) > M + Cam (47.1 ± 17.1 seconds) > M + Mon + Cam (33.4 ± 10.3 seconds) > C (20.5 ± 3.5 seconds). (multivariable analysis of variance (MANOVA), *p* = 7.9 × 10^−7^).

## Discussion

In general, the surgeon stands on the right side of the patient and the assistant stands on the left side of the patient during laparoscopic left-sided colorectal surgery. The camera is placed through the umbilical port. The monitor for the surgeon is in front of the surgeon. The monitor for the assistant is in front of the assistant. A third monitor is placed by the patient’s legs.

During the parts of the procedure involving the rectum, the surgeon and the assistant look at the monitor by the patient’s legs. When lymph node dissection around the IMA is performed, both look at the monitor in front of them. The assistant usually encounters a mirror image during dissection around the IMA because the camera and the direction of the assistant’s line of sight are aligned in reverse orientation^1,2,5^.

With a mirror image, the actual view and the view on the monitor differ by 180 degrees^2,9^. It took a long time for study participants to perform the task with a mirror image because they might be required to mentally rotate the image by 180 degrees. Several studies in cognitive neuroscience research have reported that the time to correct the direction of a rotated image increases as the angle of rotation increases^13–15^. In the present study, the time for completing the task with a mirror image was widely distributed, possibly because the participants might have different amounts of surgical experience^5^.

When the monitor was placed on the participant’s left side with mirror image or the camera was rotated 90 degrees to the right with mirror image, it seemed that the view on the monitor was rotated 90 degrees to the left for the participant. Task performance with a mirror image improved, because the difference in angle between the actual view and the view on the monitor seemed to be reduced to 90 degrees.

When the monitor was placed on the left side and the camera was rotated 90 degrees to the right, it seemed that the view on the monitor was rotated 180 degrees to the left for the participant. Task performance with mirror image improved remarkably, because the difference in angle between the actual view and the view on the monitor seemed to be reduced to 0 degrees. In the present study, the time for completing the task in this situation was narrowly distributed, because the participants might have overcome the challenge posed by a mirror image regardless of surgical experience.

When the young inexperienced assistant surgeon encounters a mirror image during actual laparoscopic colorectal surgery, the assistant looks at the monitor placed by the patient’s legs (i.e., on the assistant’s left side) and the camera is rotated 90 degrees to the right. Then, the assistant can perform relatively well with mirror images. Once the assistant supports the operative field, the assistant does not need to move too much. At this time, the camera can be rotated back to the normal angle and the surgeon can begin the procedure. Using this novel method, laparoscopic left-sided colorectal surgery might be performed more safely and smoothly. This method might be useful for left-sided colorectal resection in laparoscopic surgery of deep infiltrating endometriosis during gynecological procedures^16,17^.

The present study had several limitations. First, this method was not evaluated during actual laparoscopic surgery due to ethical issues. Second, we did not investigate whether performance with mirror images improved with other monitor positions and camera rotation angles. Further studies are needed.

The present study was the first study to investigate optimal monitor positioning and camera rotation angle for mirror images. The mirror image problem in laparoscopic surgery might be closely related to the mental rotation problem in cognitive neuroscience. Changing monitor positioning and camera rotation angle improved task performance with mirror images by reducing the difference in angle between the actual view and the view on the monitor. This novel method might be a simple way to overcome mirror image in laparoscopic left-sided colorectal surgery.

## Methods

**Figure 2:**
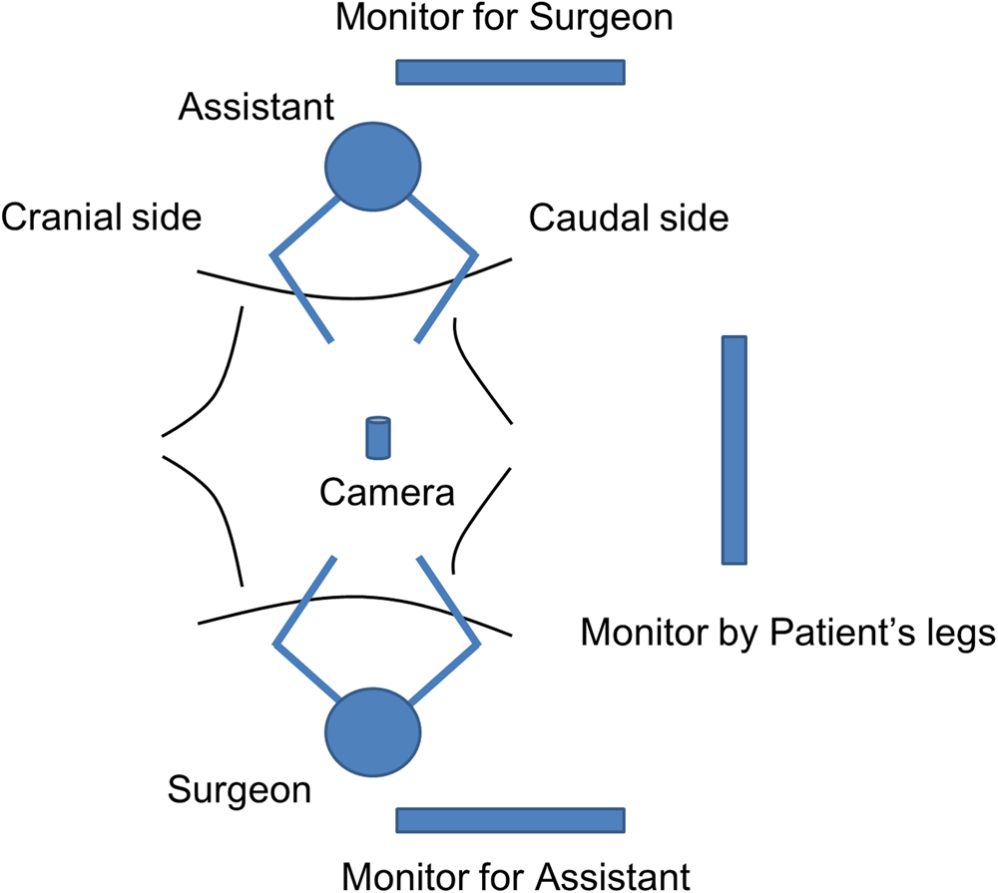
Usual setup for laparoscopic left-sided colorectal surgery. The surgeon usually stands on the right side of the patient and the assistant stands on the left side of the patient. The camera is placed through the umbilical port. The monitor for the surgeon is in front of the surgeon. The monitor for the assistant is in front of the assistant. A third monitor is placed by the patient’s legs.

The present study was set up to investigate whether: (1) positioning the monitor on the left side improves task performance with a mirror image, (2) rotating the camera 90 degrees to the right improves task performance with a mirror image, and (3) simultaneously positioning the monitor on the left side and rotating the camera 90 degrees to the right improves task performance with a mirror image. The monitor was positioned on the left side because the monitor is placed on the left side of the assistant (by the patient’s legs) during actual left-sided laparoscopic colorectal surgery (Fig. 2). We investigated a camera rotation angle of 90 degrees to the right because 90 degrees of rotation might be permissible for an experienced surgeon^13^. These methods were carried out in accordance with the relevant guidelines and regulations with regards to privacy protection of the participants. These experimental protocols were consulted with the Ethical Committee in Kobe University Hospital. The Ethical Committee decided that it was not necessary to discuss with these protocols in the committee, because the participants were not patients, but surgeons. These protocols were approved by Academic Research Committee in Department of Surgery, according to the advice of the Ethical Committee.

### Participants

Twenty-four surgeons in postgraduate years 4–9 who did not have sufficient surgical experience with mirror images participated in the present study. Informed consent was obtained from all participants.

### Task

**Figure 3:**
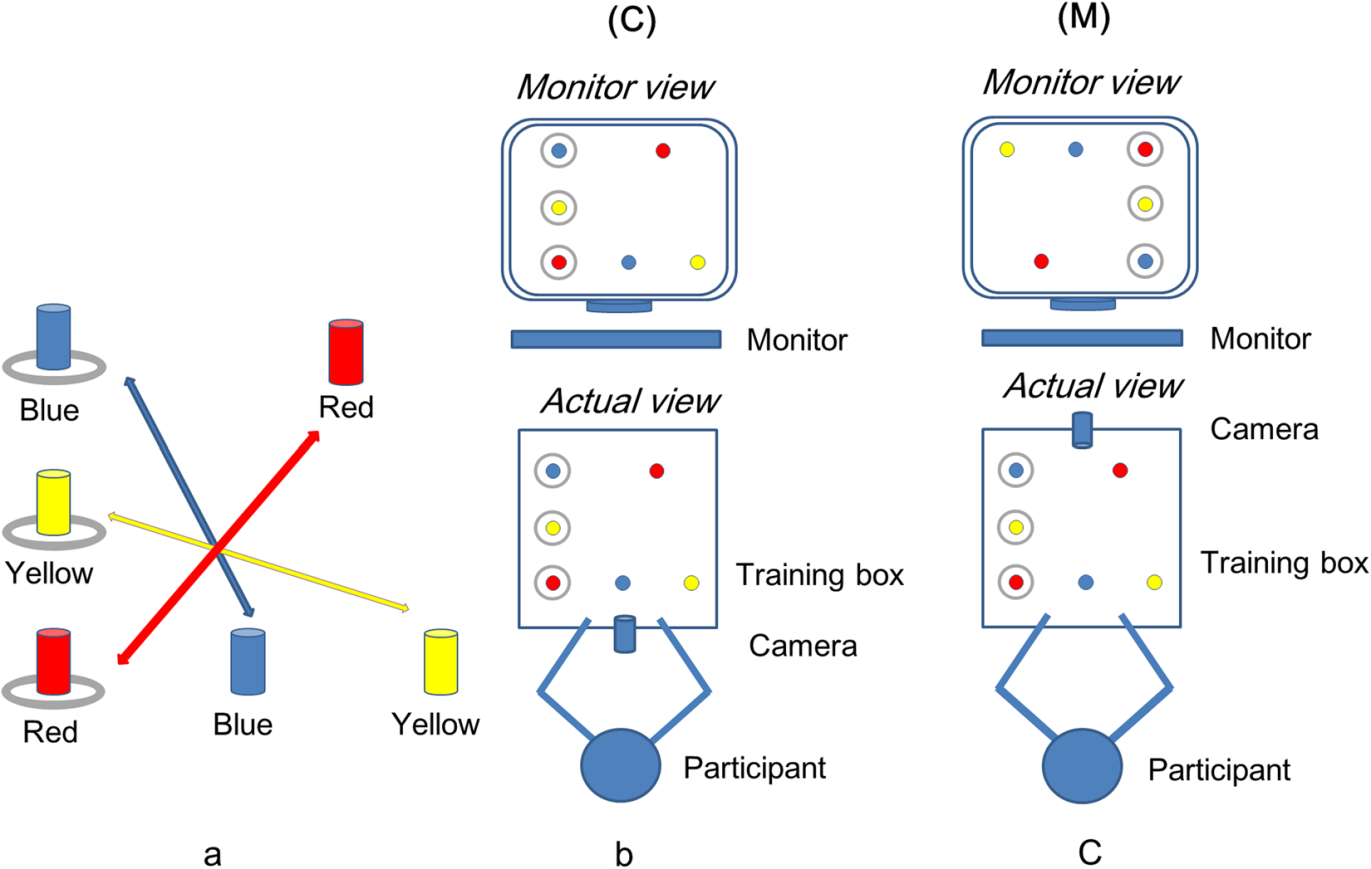
Setup of the training box. (**a**) Modified peg transfer task: There are three pegs in blue, yellow, and red, on the left side of the pegboard in a straight line and three other pegs in corresponding colors on the right side of the pegboard in a triangle. Using laparoscopic forceps, the participants moved three ring shaped objects from the left side to the right side while matching colors, and moved the three ring shaped objects back from the right side to left side while matching colors. (**b**) Coaxial image (C): the camera faced the target in the direction of the participant’s line of sight and the monitor was in front of the participant. The angle of the view on the monitor was the same as the actual view. (**c**) Mirror image (M): the camera was placed on the opposite side of the participant. The difference in angle between the actual view and the view on the monitor was 180 degrees.

The peg transfer task was adopted because it includes basic required manipulations such as grasping, moving, and releasing^18,19^. The assistant usually performs these manipulations during laparoscopic colorectal surgery. However, the peg transfer task in the Fundamentals of Laparoscopic Surgery (FLS) module was difficult for participants to accomplish with a mirror image. Thus, we developed a modified peg transfer task (Fig. 3a), which is a simplified version of the FLS peg transfer task.

In the modified peg transfer task, the pegboard is placed in the middle of the training box. There are three pegs in blue, yellow, and red, on the left side of the pegboard in a straight line and three other pegs in corresponding colors on the right side of the pegboard in a triangle (Fig. 3a). The participants were asked to use laparoscopic forceps to move the three ring shaped objects from the left side to the right side while matching colors using the left hand, and to move the three ring shaped objects back from the right side to left side while matching colors using the right hand.

### Setup

**Figure 4:**
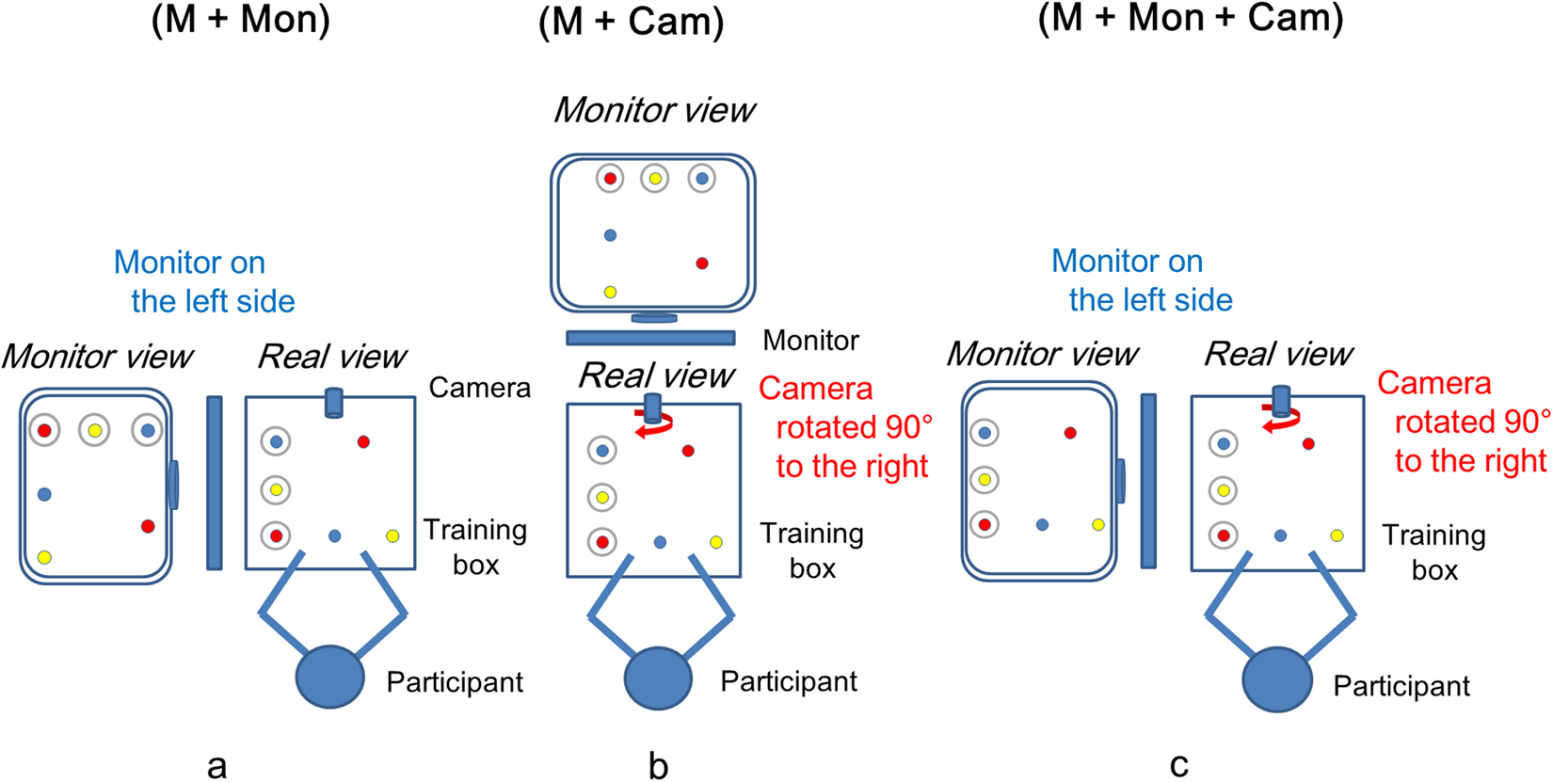
Setup of the training box for the experimental conditions. (**a**) Mirror image + Monitor on the left side (M + Mon): the monitor was placed on the left side of the participant in mirror image. The difference in angle between the actual view and the view on the monitor seemed to be reduced to 90 degrees. (**b**) Mirror image + Camera rotated 90 degrees to the right (M + Cam): the camera was rotated 90 degrees to the right in mirror image. The difference in angle between the actual view and the view on the monitor was reduced to 90 degrees. (**c**) Mirror image + Monitor on the left side + Camera rotated 90 degrees to the right (M + Mon + Cam): the monitor was placed on the left side of the participant and the camera was rotated 90 degrees to the right in mirror image. The difference in angle between the actual view and the view on the monitor seemed to be reduced to 90 degrees.

We created five different conditions (Fig. 3, Fig. 4): Coaxial image (C), in which the camera faced the target in the direction of the participant’s line of sight and the monitor was in front of the participant; Mirror image (M), in which the camera was placed on the opposite side of the participant; Mirror image + Monitor on the left side of the participant (M + Mon); Mirror image + Camera rotated 90 degrees to the right (M + Cam); and Mirror image + Monitor on the left side of the participant + Camera rotated 90 degrees to the right (M + Mon + Cam) with the training box (Endowork-Pro II, KARL STORZ SE & Co, Tuttlingen, Germany). The camera was placed 15 cm from the target. The monitor was placed 100 cm from the eyes of each participant.

### Procedures

The twenty-four participants performed the modified peg transfer task five times under each of the five conditions. The order of the five conditions was randomly selected for each participant. The time to compete the task was measured in seconds from the time that the forceps were visible on the monitor. The mean time for completing the task under each condition was recorded for each participant and then averaged over all participants. The outcome measure was the mean time (mean ± standard deviation) for completing the task under each condition for all participants.

### Statistical analysis

The data was analyzed using and the paired t-test for comparisons between two groups and multivariable analysis of variance (MANOVA) for comparisons among all groups. JMP® version 14 statistical software (SAS Institute Inc., Cary, NC, USA) was used for statistical analysis. The Shapiro-Wilk test was used for normality test. The statistical significance was set at 5%. The datasets generated in present study are available from the corresponding author on reasonable request.
